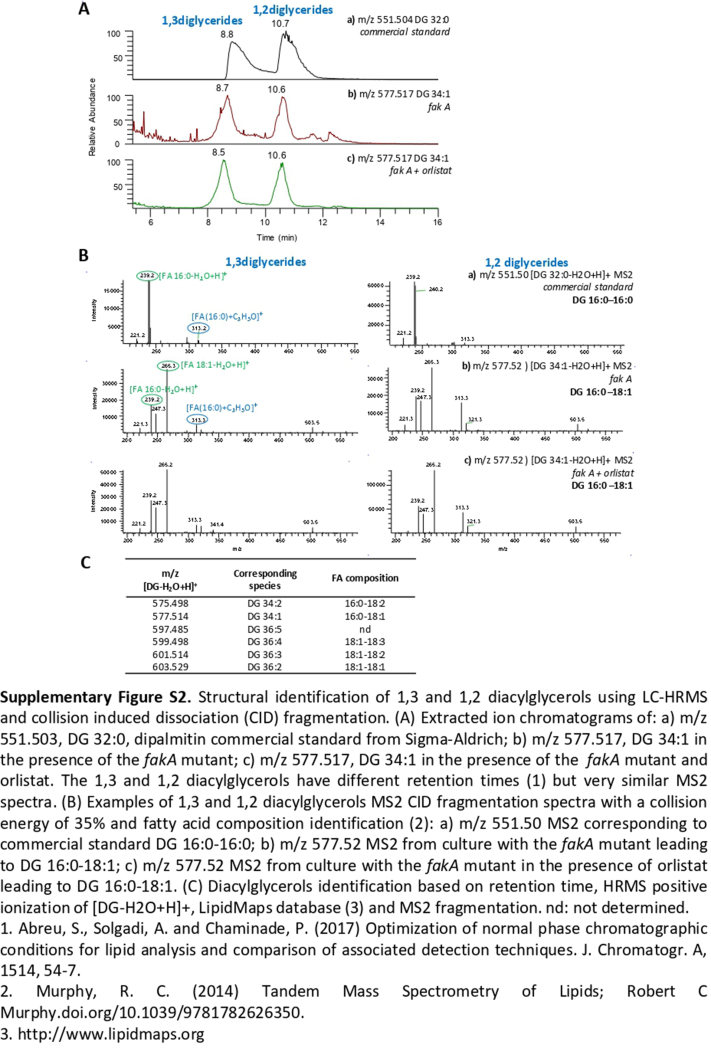# Prophages divert *Staphylococcus aureus* defenses against host lipids

**DOI:** 10.1016/j.jlr.2024.100693

**Published:** 2024-11-05

**Authors:** Biyang Zhou, Amit Pathania, Deepak Pant, David Halpern, Philippe Gaudu, Patrick Trieu-Cuot, Andressa Dias-Leao, Charlotte Pagot, Audrey Solgadi, Alexandra Gruss, Karine Gloux

**Affiliations:** 1 Université Paris-Saclay, INRAE, AgroParisTech, Micalis Institute, Jouy en Josas, France; 2 Institut Pasteur, Université Paris Cité, CNRS UMR 2001, Unité de Biologie des Bactéries Pathogènes à Gram-Positif, Paris, France; 3 UMS-IPSIT SAMM Facility, Université Paris-Saclay, Inserm, CNRS, Ingénierie et Plateformes au Service de l'Innovation Thérapeutique, Paris-Saclay, France

**Keywords:** fatty acid transport, phospholipases C, triglycerides, sphingolipids, lipolysis and fatty acid metabolism

## Abstract

Phages are ubiquitous in bacteria, including clinical *Staphylococcus aureus*, where Sfi 21/Sa3 phages often integrate into the *hlb* gene, which encodes Hlb sphingomyelinase. This integration acts as a rapid regulatory switch for Hlb production. Our findings suggest that Sfi 21/Sa3 prophages and Hlb activity influence *S. aureus* fitness by modulating the incorporation of the toxic linoleic acid (C18:2) from serum into the bacterial membrane. This process relies on C18:2 derived from 1,3-diglyceride, facilitated by the FakB1 kinase subunit. Palmitic acid (C16), primarily released from serum through Hlb activity, competes with C18:2 for FakB1. This mechanism contributes to adaptation to AFN-1252, an antibiotic inhibiting the fatty acid synthesis pathway (anti-FASII). Since *S. aureus* relies on exogenous fatty acids for growth, AFN-1252 treatment leads to an increased proportion of C18:2 in the membrane. Furthermore, Hlb inhibition, whether by prophage insertion, gene inactivation, or enzyme inhibition, delays *S. aureus* adaptation, resulting in a higher proportion of C18:2 in the membrane. This study sheds light on the role of lipid environments in infections and may contribute to the accurate prediction of infection risks and therapeutic efficacy. Moreover, since both anti-FASII agent and Hlb inhibitor enhance C18:2 incorporation, they represent potential candidates for combined strategies against *S. aureus*.

Lipids are crucial for bacterial fitness and play a pivotal role in modulating interactions with the host (1, 2, 3, 4). However, the impact of environmental lipids on bacterial pathogenesis is often overlooked. Lipids range in complexity from simple short hydrocarbon chains to complex molecules such as triglycerides (TGs), phospholipids (PLs), esterified sterols, and sphingolipids. Fatty acids (FAs), the fundamental building blocks of complex lipids, vary in chain length, side chains, and the number and positions of double bonds. In humans, pathogens encounter diverse lipid environments influenced by genetics and diet, which can correlate with infection risk (2, 3, 5, 6). Bacterial lipases break down host-derived lipids into free fatty acids (FAs), which many bacteria can scavenge and incorporate into their membranes (7, 8, 9, 10, 11, 12). *Staphylococcus aureus*, a major human pathogen, is particularly adept at incorporating exogenous FAs, such as those found in serum lipids (8, 13). Once liberated, these FAs are incorporated into the bacterial membrane through the FakAB system, consisting of FakA (an FA kinase) and the binding proteins FakB1 and FakB2, which prefer saturated and unsaturated FAs, respectively (14, 15, 16). Since *S. aureus* lacks desaturase enzymes and cannot synthesize unsaturated FA (17), it relies on external sources for these compounds. The incorporation of monounsaturated (MUFAs) and polyunsaturated FAs (PUFAs) significantly impacts bacterial membrane functions and influences interactions with the host (18, 19, 20).

Prophages of the Sfi 21/Sa3 family are the most prevalent phages integrated into staphylococcal genomes, present in over 90% of human clinical isolates (21, 22, 23, 24, 25). These prophages have an insertional hotspot in the *hlb* gene (*hlb*-conversion), which encodes the *S. aureus* neutral sphingomyelinase C (Hlb, EC 3.1.4.12, also known as hemolysin B), an enzyme that hydrolyzes sphingomyelins (Fig. 1A) (21, 24). The significance of Sfi 21/Sa3 prophages for *S. aureus* adaptation to the human host is attributed to the immune evasion cluster (IEC) encoded by the prophage, as well as to the regulation of Hlb activity (26, 27). Sfi 21/Sa3-prophages act as active lysogens, meaning they can excise from the *hlb* gene in response to environmental signals, such as biocides or reactive oxygen species, without killing the bacteria (26, 27). The released phage DNA (termed episome) can facilitate rapid adaptation through reinsertion into the same site. Although the roles of sphingomyelinases and IEC in virulence are well studied ((25, 28), for review), the impacts of these prophages on *S. aureus* physiology are less understood. Moreover, Sfi 21/Sa3-prophages are implicated in host switching between animals and humans (29, 30), making their physiological impact on bacteria crucial for a One Health approach to *S. aureus.*

**Fig. 1:**
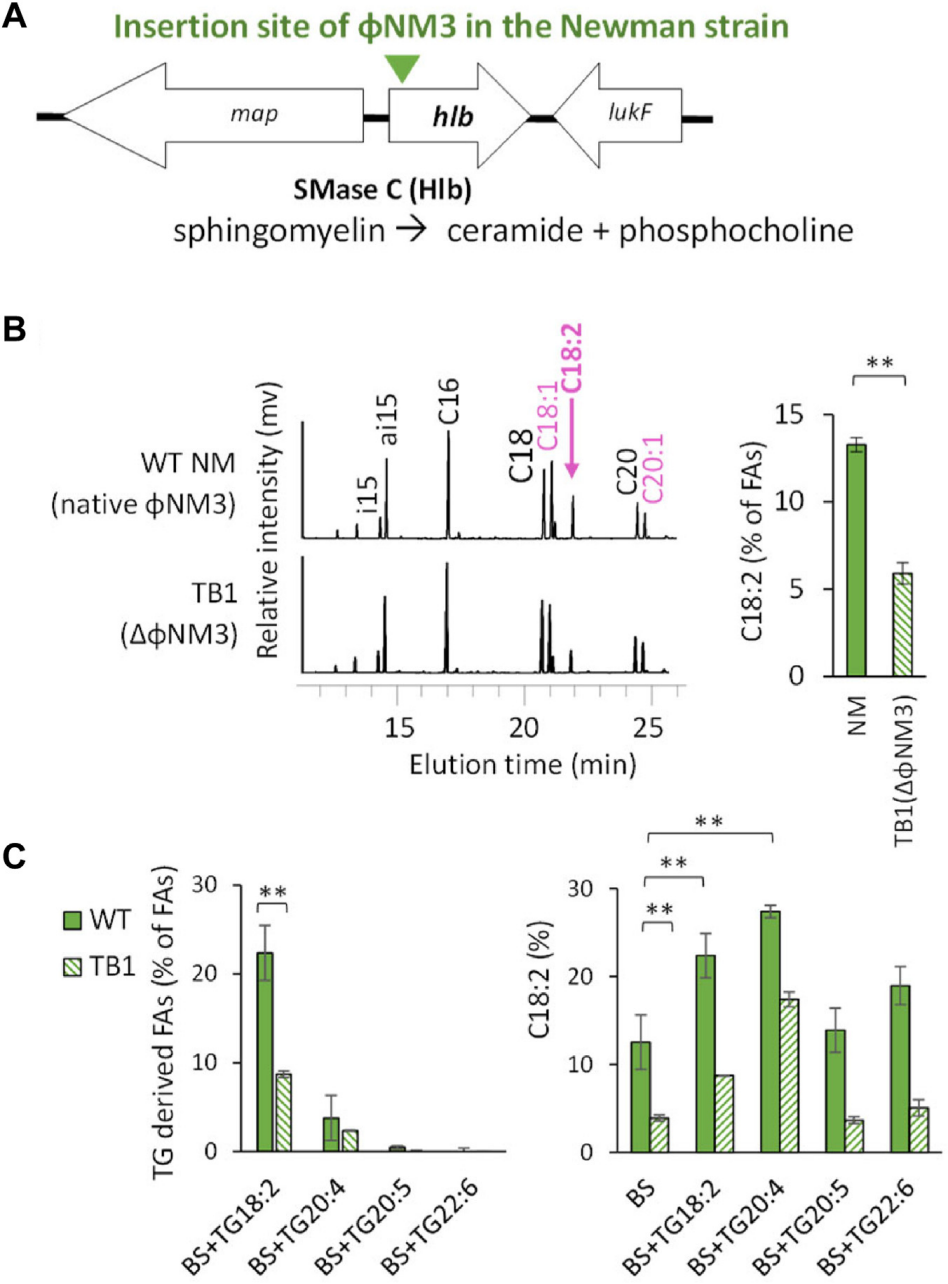
*hlb*-converting prophage enhances incorporation of linoleic acid (C18:2) from serum and triglycerides (TGs). A: The insertion site of phage ɸNM3 in the *hlb* gene, which encodes the enzyme sphingomyelinase Hlb, results in a truncation at the enzyme’s N-terminus at position 56 of the 330 amino acid sequence. The gene *map* encodes an extracellular adherence protein of broad specificity (Eap/Map); and the gene *lukF* encodes a protein from the leukocidin/hemolysin toxin family. B: The *hlb*-conversion increases the incorporation of C18:2 from mouse serum. The wild-type Newman strain (WT NM), containing the *hlb*-converting ɸNM3 prophage, and the TB1 strain, lacking ɸNM3 and having an intact *hlb* gene, were grown for 2 h in BHI medium supplemented with 10% mouse serum. FAs were extracted from PLs and analyzed by gas chromatography. On the left, representative FA profiles are shown with y-axis representing the relative FA abundance (mV response) at indicated peak positions. FAs that can be synthesized by *S. aureus* are shown in black, while FAs that can only be incorporated from serum are in purple. On the right, histograms depict the relative amounts of C18:2 incorporated into PLs. C: The *hlb*-conversion specifically affects the incorporation of C18:2 from TGs into PLs. The WT NM and TB1 strains were cultured in BHI medium supplemented with 10% bovine serum (BS) and enriched with 30 μM of triarachidonin (TG20:4), trieicosapentaenoin (TG20:5) or tridocosahexaenoin (TG22:6). Data are presented as mean ± standard deviation from independent experiments (n = 4 for (A), n = 3 for (B)). Statistical significance was determined using the Mann Whitney test for incorporated FAs. ∗∗, *P* ≤ 0.01.

In this study, we observed that the interruption of the *hlb* gene by the φNM3 prophage (from the Sfi 21/Sa3 family) results in increased incorporation of the bactericidal PUFA linoleic acid (C18:2) into the membrane PLs of *S. aureus*. We investigated the mechanism by which the presence of an *hlb*-converting prophage affects the incorporation of C18:2, which is primarily released from TGs (31). Our findings demonstrate that an intermediate of TG hydrolysis leads to the high incorporation of C18:2 via the FakB1 protein subunit. This unexpected binding to FakB1 results in competition with the C16 FA released from sphingomyelins for incorporation into PLs. Furthermore, we showed that inhibition of FASII and/or Hlb activities exacerbates the incorporation and toxicity of C18:2 in PLs, reducing *S. aureus* fitness.

## Materials and methods

### Bacterial strains

*S. aureus* USA300 FPR3757 (methicillin-resistant, referred to as USA300), and the NCTC 8325 derivative strains RN450 and RN450-R, were used in this study (Table 1). RN450, also designated as 8325-4, is a phage-free derivative of the NCTC 8325 strain (35) and has a deficient *fakB1* gene due to a natural deletion. RN450-R is a derivative of RN450 in which *fakB1* has been repaired, as described below. Isogenic Newman strains differentiated by the presence of prophages, referred to as WT NM (wild type), TB3 (Δɸ11), TB1 (ΔɸNM3), and TB4 (Δɸ11, ΔɸNM3), were provided by the Schneewind laboratory (Department of Microbiology, University of Chicago). The WT NM contains four prophages, including ϕNM3 from the Sfi 21/Sa3 subfamily inserted in *hlb*, and ϕNM4 inserted in *geh*, which encodes a TG lipase (21). The TB3 strain contains only the *hlb*-converting prophage ϕNM3. The Nebraska USA300 transposon insertion library (the University of Nebraska Medical Center) was generously supplied by BEI resources (36). The derivatives used in this study contained insertions, verified by PCR, in the following genes: *SAUSA300_0320* (*geh*), *SAUSA300_1973* (*hlb*), *SAUSA300_1119* (*fakA*), *SAUSA300_0733* (*fakB1*), *SAUSA300_1318* (*fakB2*). The different strains used, along with their *hlb* and *geh* status (intact or interrupted genes), and prophage insertions, are presented in Table 1.

**Table 1:** Strains and phages

Strains	Designation	*hlb* Interruption	*fakAB* Defect	*geh* Interruption	Ref
Wild types					
NM	Newman	yes; by φNM3	no	yes by φNM4	(32)
USA300	USA300_FPR3757	yes; by φSa3USA300	no	no	(33, 34)
RN450	RN0450	no	yes; deleted region in *fakB1*	no	(35)
Impact of Hlb and Geh lipases					
TB1	Newman-derived ΔφNM3	no	no	yes by φNM4 prophage	(32)
TB3	Newman-derived ΔφNM1.2.4	yes; by φNM3	no	no	(32)
TB4	Newman-derived ΔφNM1.2.4ΔφNM3	no	no	no	(32)
NE1261	USA300-derived *hlb*::tn	yes; by φSa3USA300 and Tn	no	no	(36)
NE1261p∅	USA300-derived *hlb*::tn pAW8	yes; by φSa3USA300 and Tn	no	no	this study
NE1261p*hlb*	USA300-derived *hlb*::tn pAW8*hlb*	yes; by φSa3USA300 and Tn	no	no	this study
NE1775	USA300-derived *geh*::tn	yes; by φSa3USA300	no	yes; by Tn	(36)
Role of the FakAB system in C18:2 toxicity					
USA300*fakA*	USA300-derived NE229	yes; by φSa3USA300	yes; *fakA* interrupted by Tn	no	(36)
USA300*fakB1*	USA300-derived NE1540	yes; by φSa3USA300	yes; *fakB1* interrupted by Tn	no	(36)
USA300*fakB2*	USA300-derived NE403	yes; by φSa3USA300	yes; *fakB2* interrupted by Tn	no	(36)
RN450-R	RN0450-R	no	no; repaired for *fakB1*	no	this study

### Construction of an *hlb* complemented strain

A PCR fragment containing the *hlb* promoter and the open reading frame SACOL_RS10470 (−355 from the ATG start codon and +40 after the TAG stop codon) from the *S*. *aureus* subsp. *aureus* COL, was cloned between EcoRI and SmaI sites of plasmid pAW8 (37). The primer pairs used for the PCR were 5′-TTGCCGGAATTCTGCAACTTAATTATAGCCAGACTTTC-3′ and 5′-CATCAACCCGGGCGTCCTTTTAGAACGAAGCAAG-3'. Genomic coordinates of the 1388 bp cloned segment were 2063371–2064758. This plasmid, named p*hlb*, was used for complementation experiments.

### Construction of the RN450-R strain, repaired for *fakB1*

**Supplementary Figure S1:**
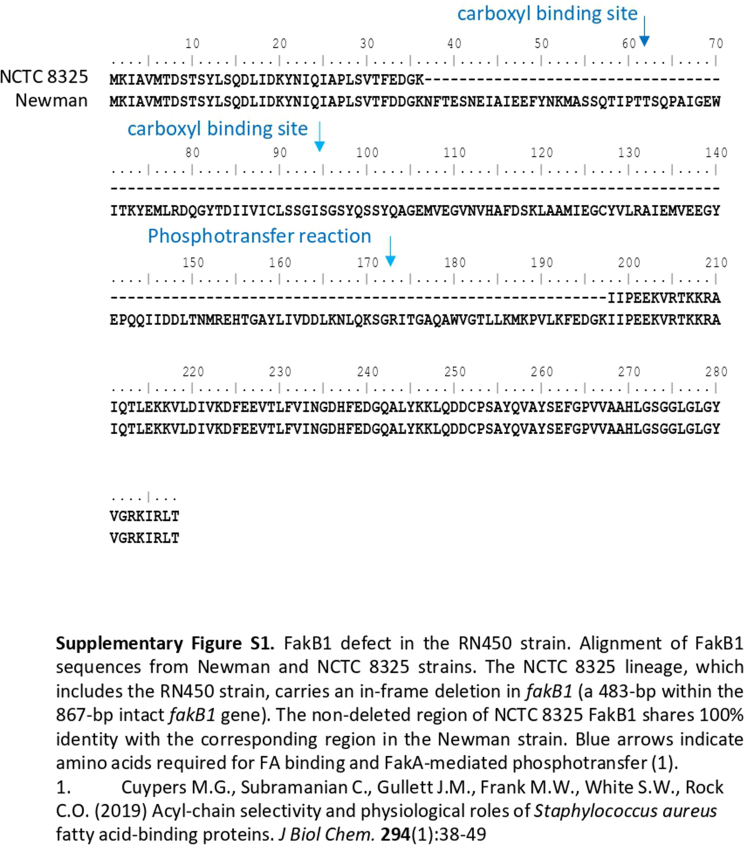


In the RN450 strain, the *fakB1* gene lacks a 483-bp internal segment. This deletion is throughout the entire NCTC 8325 lineage, removing 56% of the 867-bp functional gene (supplemental Fig. S1). To repair this deletion, a 1,939-bp DNA fragment containing a functional *fakB1* was amplified to restore the function in RN450, as described for other NCTC 8325 derivatives (38).

### Growth media and conditions

Brain heart infusion (BHI) was used as the base medium for *S. aureus* cultures. Bacteria were grown aerobically at 37°C with a starting OD_600_ of 0.02, from 2 to 5-h precultures prepared in BHI. Mouse or adult bovine serum (Clinisciences, France) was added at 10% final concentration. Stocks of trilinolein (TG18:2, 50 mM), triarachidonin (TG20:4, 10 mM), trieicosapentaenoin (TG20:5, 50 mM), tridocosahexaenoin (TG22:6, 50 mM), 1,2-dilinoleoylglycerol (1,2DG, 50 mM), 1,3-dilinoleoylglycerol (1,3DG, 50 mM), palmitic acid (C16, 100 mM), (Larodan Fine Chemicals), orlistat (38 mM, MedChemExpress), and the anti-FASII antibiotic AFN-1252 (1 mg/ml, MedChemExpress) were prepared in DMSO.

In the absence of an anti-FASII, FA profiles, and C18:2 incorporation were determined from bacteria recovered after short growth times (OD_600_ between 1 and 4) to avoid complete consumption of C18:2. Bacterial growth is thus presented as the variation of OD_600_ per hour (OD_600_/h). To monitor *S. aureus* adaptation to an anti-FASII, bacteria were pre-cultured for 4 h in BHI medium supplemented with 10% mouse serum (Eurobio) and inoculated at OD_600_ 0.02 in the same medium containing 0.5 μg/ml of the anti-FASII AFN-1252. Bacteria were cultured for 17 h at 37°C in a 96-well microtiter plate using a Spark spectrophotometer (Tecan), with OD_600_ measured every 10 min. Growth results are presented as bacterial growth kinetics over time.

### Hlb substrate, inducer, and inhibitor

Sphingomyelin was derived from chicken egg yolk (Sigma-Aldrich) and prepared as a 50 mg/ml stock solution in chloroform/methanol (1:1). Hlb sphingomyelinase belongs to the neutral sphingomyelinase family, which requires a lipid-dependent activation by phosphatidylserine. Phosphatidylserine (60 mM, bovine brain, Na salt, Larodan) was provided in chloroform. GW4869 (Selleckchem.com), a specific inhibitor of the conserved domain of Hlb involved in lipid activation (39, 40), was prepared at 1.7 mM in DMSO.

### Determination of *S. aureus* fatty acid profiles

FAs profiles were performed as described previously (9). Briefly, culture aliquots were taken during exponential growth at low OD_600_ (between 1 and 4). After sample preparation, extraction, and trans-esterification of FAs, gas chromatography separation was performed by injection of the FA methyl esters in a split-splitless mode on an AutoSystem XL gas chromatograph (PerkinElmer) equipped with a ZB-Wax capillary column (30 m × 0.25 mm × 0.25 mm; Phenomenex) and a flame ionization detector. FAs were identified based on their retention times and coinjection with purified FA methyl esters standards (Mixture ME100, Larodan). Data were recorded and analyzed using a TotalChrom Workstation (PerkinElmer). FA peaks were detected between 12 and 40 min of elution. Results are shown as representative gas chromatograph profiles or as FA peak areas expressed as a percentage of the total areas of detected peaks.

### Detection and characterization of diglycerides, ceramides, and sphingomyelins

Lipid extractions were performed as described by E.G. Bligh and W.J. Dyer (41) with modifications (42). Briefly, freeze-dried supernatants (10 ml) of culture (OD_600_ from 3 to 4) were extracted with 9.5 ml of chloroform-methanol 0.3% NaCl (1:2:0.8 v/v/v) at 80°C for 15 min and vortexed for 1 h at room temperature (RT). After centrifugation at 4,000 rpm for 15 min (RT) supernatants were collected and the debris were re-extracted with 9.5 ml of the same mixture and vortexed for 30 min. After another centrifugation, supernatants were pooled, and 2.5 ml of each chloroform and 0.3% NaCl solution were added and mixed. Phase separation was achieved by centrifugation at 4,000 rpm for 15 min (RT). The upper phase was discarded, and the chloroform phase was evaporated to dryness under a nitrogen stream and stored at −20°C. Lipids were identified using a previously described method (43). Sphingomyelins and ceramides were identified and characterized by coupling normal phase liquid chromatography with mass spectrometry (NPLC-MS) in negative and positive atmospheric pressure chemical ionization modes (APCI−/+), respectively, with species confirmed by collision-induced dissociation MS^2^/MS^3^ fragmentations (LTQ-Orbitrap Velos Pro). Diglycerides species were determined as illustrated in supplemental data (supplemental Fig. S2). Briefly the identification of 1.2- and 1.3- diglycerides was performed from retention times, through comparison with pure diglyceride standards, and the extracted ion current of the main species. The molecular formula of the 1,3-diglycerides, including the number of carbons and the degree of unsaturation, were identified by HRMS, LipidMaps database, and MS2 fragmentation. Diglycerides were ionized in positive APCI with a loss of water and the addition of a proton [DG-H_2_O + H]^+^. Lipid spectra were analyzed using Xcalibur™ software (ThermoFisher Scientific, version 4.2.47).

### Statistics analysis

Graphs were prepared using Microsoft Excel. Means and standard deviations are presented for culture growth (OD_600_ or OD_600_/h), FA percentages, and percentages of FA elongated via FASII. Statistical significance was determined using unpaired, nonparametric Mann-Whitney tests, as recommended for small sample sizes. For dependent samples, the non-parametric Wilcoxon Signed Rank test was applied.

## Results

### The *hlb*-converting prophage ɸNM3 increases C18:2 incorporation from serum into *S. aureus* phospholipids

Two isogenic Newman strains of *S. aureus*, WT and TB1, differing by the presence of the *hlb*-converting prophage ɸNM3 (Table 1) (32), were grown in the control BHI medium. FAs present in their PLs were extracted and analyzed. Both strains exhibited identical FA profiles, corresponding to endogenous FAs produced by FASII activity (supplemental Fig. S3), indicating that the ɸNM3 prophage does not affect FAs in membrane PLs in this culture condition. However, supplementation with 10% mouse serum led to the incorporation of exogenous PUFAs, which *S. aureus* cannot synthesize (17). Notably, only the proportion of C18:2 in PLs was significantly affected by the presence of ɸNM3, showing a two-fold increase in the WT strain compared to TB1 (Fig. 1B). To confirm this observation, we tested adult bovine serum, which contains a broader spectrum of PUFAs, including C18:2, α-linolenic acid (C18:3 ω-3), dihomo-γ-linolenic acid (20:3 ω−6), and arachidonic acid (C20:4 ω-6) (supplemental Fig. S4). Since various PUFAs are commonly found in the human body, primarily transported as TGs, we also enriched cultures with different TGs, each containing a single distinct long-chain PUFA commonly found in vivo: C18:2, C20:4 ω-6, eicosapentaenoic acid (C20:5 ω-3), or docosahexaenoic acid (C22:6 ω-3). FA profiles of the WT and TB1 strains grown with these PUFAs revealed that only two PUFAs were incorporated into *S. aureus* PLs: C18:2 and, to a much lesser extent, C20:4 (Fig. 1C). Furthermore, the presence of ɸNM3 mainly affected C18:2 incorporation (Fig. 1B and C). Enrichment with 30 μM trilinolein (TG18:2 in Fig. 1C), a TG source containing three C18:2 molecules, further increased the proportion of C18:2 in PLs in both strains, indicating that the prophage’s effect persisted even with enrichment. Interestingly, enrichment with triarachidonin (TG20:4), a TG source of C20:4, also increased C18:2 incorporation in both strains, to an even greater extent than TG18:2 (Fig. 1C). Although the exact mechanism remains unclear, these results suggest that the prophage specifically modulates C18:2 incorporation into the membrane PLs of *S. aureus*. This effect is unexpected, as serum sphingomyelins are poor sources of C18:2 (18, 44). We therefore questioned how ɸNM3 is linked to C18:2 incorporation into *S.aureus* PLs.

### C18:2 from 1,3-dilinoleoylglycerol (1,3 DG) is highly incorporated and toxic in *S. aureus*

**Fig. 2:**
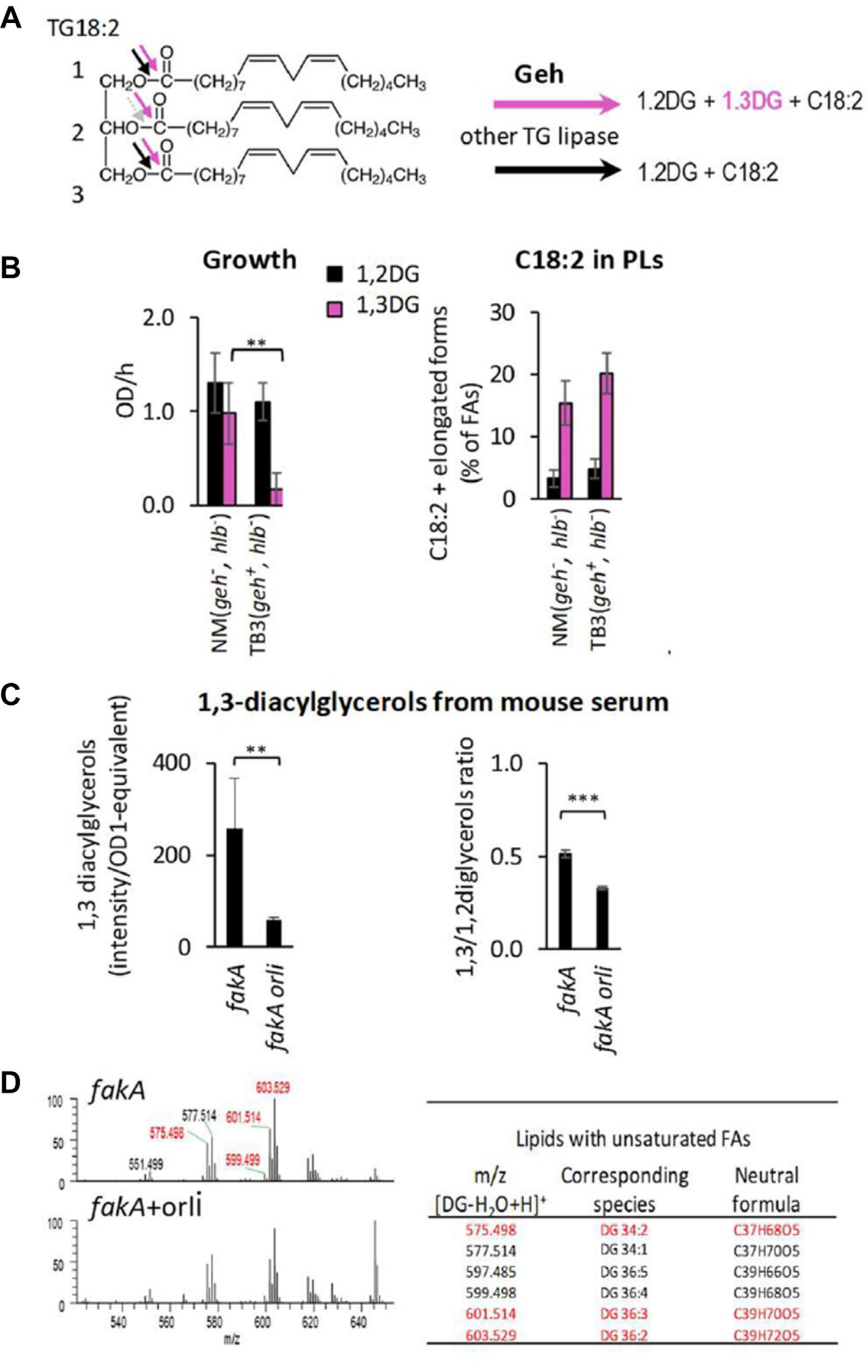
Crucial role of 1,3-diacylglycerols in C18:2 toxicity in *S. aureus*. A: The non-selective cleavage of TGs by *S. aureus* Geh activity results in both 1,2- and 1,3- diacylglycerols (1,2DG and 1,3DG from TG18:2). Most lipases preferentially cleave at position 1, releasing 1,2-diacylglycerols, as illustrated for 1,2DG from TG18:2. In contrast, Geh can cleave at both positions, thus releasing 1,3-diacylglycerols (45, 46). B: The 1,3DG supplementation results in a high incorporation of C18:2 into PLs. WT NM (*geh*::tn) and TB3 (intact *geh*) strains were cultured for 2 h in BHI medium supplemented with 10 μM of either 1,2DG or 1,3DG. The relative amounts of incorporated C18:2 (C18:2 and its elongated forms) were determined as in Figure 1B and are presented as FAs derived from the diacylglycerols. Notably, C18:2 was more efficiently incorporated and toxic when derived from 1,3DG compared to 1,2DG, with TB3 (intact *geh*) showing extreme sensitivity. C: *S. aureus* lipase generates 1,3-diacylglycerols. The USA300 *fakA*::tn mutant was cultured in BHI medium with 10% mouse serum, with or without 30 μM orlistat, an inhibitor of the Geh activity. Lipids present in the supernatant were extracted and analyzed using normal phase liquid chromatography (NPLC) coupled with high-resolution mass-spectrometry (HRMS). 1,2 and 1,3 diacylglycerols were identified by their retention times and through comparison with pure diacylglycerol standards (supplemental Fig. S2). D: The 1,3-diacylglycerols generated by *S. aureus* lipase contain polyunsaturated FAs. The 1,3-diacylglycerols with polyunsaturation (formulas in red) and generated in (C) were identified by HRMS. They were ionized in positive APCI with a loss of water and the addition of a proton [DG-H_2_O + H]^+^ (supplemental Fig. S2). Data are presented as mean ± standard deviation from independent experiments (n = 4 for (B), n = 3 for (C)). Statistical significance was determined by the Mann–Whitney test in (B) and by the non-parametric Wilcoxon Signed Rank test in (C). ∗∗, *P* ≤ 0.01. ∗∗∗, *P* ≤ 0.001.

*S. aureus* triglyceride lipases can produce both 1,2- and 1,3- diacylglycerol intermediates from TG hydrolysis, as illustrated by the TG18:2 example (Fig. 2A). We analyzed the impact of 1,2-dilinoleoylglycerol (1,2DG) and 1,3-dilinoleoylglycerol (1,3DG) on *S. aureus* growth and their efficiency as substrates for C18:2 incorporation into PLs (Fig. 2B). Since 1,3DG is preferentially released by Geh lipase activity (45, 46) (Fig. 2A), the experiment was conducted using the USA300 *geh*^*+*^ and *geh*^*-*^ strains (Table 1). We observed that 1,3DG-derived C18:2 was highly incorporated into *S. aureus* PLs at a much higher rate compared to that from 1,2DG (Fig. 2B). Additionally, 1,3DG was significantly more toxic in the *geh*^+^ strain than in the isogenic *geh*^*-*^ strain (Fig. 2B). However, the resulting proportion of C18:2 and its elongated forms is similar for both strains, suggesting that it is not the determining factor for toxicity. Instead, both the hydrolysis position on the glycerol backbone and the *geh* status are crucial for the toxicity of the released C18:2. We further investigated whether 1,3-diacylglycerols could be released by *S. aureus* in the presence of serum and whether they contain PUFAs. We used a USA300 *fakA* mutant to block FA incorporation (14) and increase the likelihood of detecting 1,3-diacylglycerol intermediates. Mass spectrometry with positive APCI ionization detection identified 1,3-diacylglycerols in the supernatant after growth of the *fakA* mutant in the presence of 10% mouse serum (Fig. 2C, supplemental Fig. S2). Orlistat, an inhibitor of Geh activity (47), reduced the levels of both 1,3-diacylglycerols and the 1,3-diacylglycerols/1,2-diacylglycerols ratio in the supernatant (Fig. 2C). Characterization of the 1,3-diacylglycerols species showed that orlistat also decreased the relative abundance of polyunsaturated 1,3-diacylglycerols (Fig. 2D, supplemental Fig. S2). In conclusion, in the presence of serum, 1,3-diacylglycerol intermediates are released by bacterial lipases and serve as sources of PUFAs. More than 85% of *S. aureus* isolates are *geh*^+^ (1) and, as a result, may release polyunsaturated 1,3-diacylglycerols in the presence of serum.

### *hlb* protects *S. aureus* from 1,3DG-derived C18:2 and promotes C16 incorporation over C18:2

**Fig. 3:**
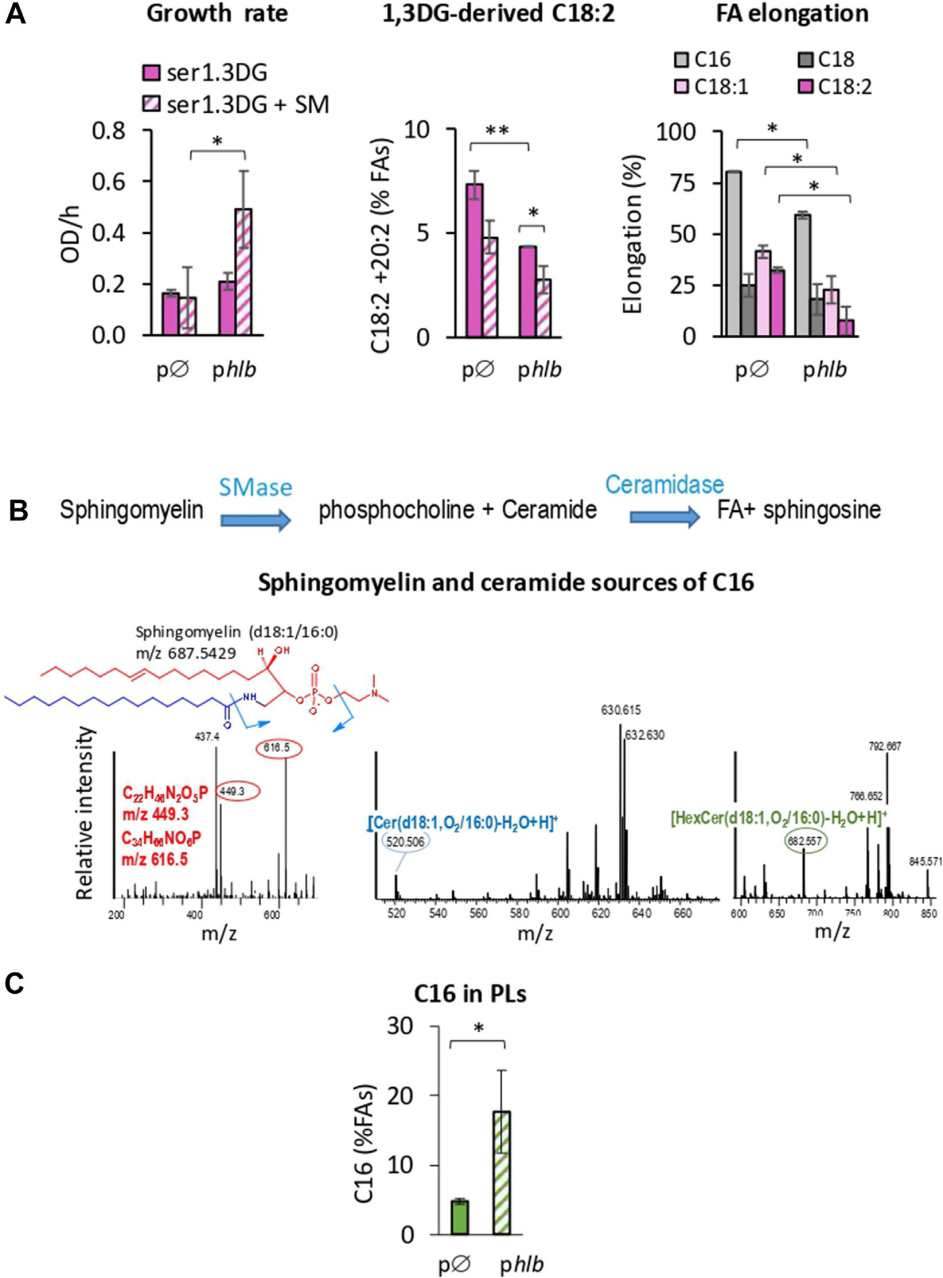
Plasmid-driven expression of *hlb* affects *S. aureus* fitness, inhibits FASII, and increases C16 in PLs. A: The *hlb*-complementation protects from the incorporation and toxicity of 1,3DG-derived C18:2 but inhibits FA elongation. The NE1261 mutant (Tn insertion in *hlb*), carrying either an empty plasmid (p∅) or a plasmid with an intact *hlb* (p*hlb*) was grown for 3 h in BHI medium supplemented with 10% mouse serum (ser) and 10 μM 1,3DG. Activated sphingomyelin (SM, 200 μM, see Materials and methods) was added as an Hlb substrate. Left: Growth is presented as the OD_600_ increase per hour. Middle: Incorporation of C18:2 and its elongated form C20:2 in PLs. Relative amounts of incorporated FAs were determined as in Figure 2B and expressed in a percentage of total FAs. Right: FA elongation by FASII was determined from the relative amounts of elongated forms of C16, C18, C18:1, and C18:2, and expressed as a percentage of the total (FA + elongated) for each. B: Sphingomyelins and ceramides from *S. aureus* culture supernatants are sources of C16. The USA300 strain was cultured in BHI medium in the presence of 10% mouse serum. Lipids were extracted, and sphingomyelins and ceramides were identified and characterized by NPLC-MS in negative and positive atmospheric pressure chemical ionization mode, respectively, with species confirmed by MS^2^ and MS^3^. Left: The main sphingomyelin species deduced from NPLC and subsequent fragmentations is SM(d18:1/16:0). The detection threshold of SM species was around 0.025 mg/ml. Traces of SM(d18:1/18:1) and SM(d18:1/18:0) were detected but no source of C18:2 was identified. Middle and Right: Ceramide intermediates containing C16 were detected in culture supernatants: a dihydroceramide (Cer(d18:1,O2/16:0)), and a hexylceramides (HexCer(d18:1,O2/16:0)). C: plasmid-driven expression of *hlb* increases C16 incorporation from sphingomyelin. Cultured NE1261 p∅ and p*hlb* strains were analyzed for FAs content as described in (A). Total C16 incorporated in PLs was determined as for C18:2 in Figure 1B and expressed as a percentage of total FAs. Data presented in histograms are means ± standard deviations from independent experiments (n = 3). Statistical significance was determined by the Mann-Whitney in (A) and (C). ∗∗, *P* ≤ 0.01, ∗*P* ≤ 0.05.

We investigated the potential impact of the *hlb* gene on C18:2 incorporation into PLs. To this end, we used the NE1261 strain, a USA300 FPR3757 derivative with an inactive *hlb* gene due to both prophage conversion and transposon insertion, and performed *hlb* complementation. A plasmid carrying an intact *hlb* (p*hlb*) from the *S. aureus* COL strain was constructed and introduced into the NE1261 strain (Table 1). NE1261p*hlb* and the control strain (NE1261pØ, empty plasmid) were grown in BHI supplemented with 10% mouse serum and 10 μM 1,3DG, with or without sphingomyelin (see Materials and methods (39)). Without sphingomyelin supplementation, *hlb* complementation had little to no effect on growth rate. Remarkably, the C18:2 incorporation into PLs was however significantly inhibited (Fig. 3A). Additionally, sphingomyelin supplementation significantly improved growth and led to poor incorporation of C18:2 from 1,3DG into PLs. Surprisingly, *hlb* complementation also significantly inhibited the elongation of both saturated and unsaturated FAs (Fig. 3B). As C18:2 was not transferred into PLs, it may remain in its free form intracellularly, potentially inhibiting the enoyl reductase enzyme (FabI) in the FASII pathway, as previously described (31). Since sphingomyelins are potential FA sources (Fig. 3B), we hypothesized that FAs could be released and compete with C18:2 for incorporation into PLs. This would likely involve an intermediate step requiring ceramidase to release such competitive FAs. Although *S. aureus* is not known to produce ceramidase, this enzyme can be present in serum (48). To confirm this, we analyzed sphingomyelin composition and searched for ceramide intermediates in the BHI-mouse serum used for cultures, as well as in the supernatant after the growth of *S. aureus* USA300. Palmitic acid (C16), a common sphingomyelin constituent in human blood (44, 49), was identified as the main FA in sphingomyelins under BHI-mouse serum conditions of our first experiment (Fig. 3B). Ceramide intermediates with C16 acyl sources were also found in culture supernatants (Fig. 3B). Furthermore, *hlb*-complementation increased C16 levels in PLs (Fig. 3C). Based on these findings, the effect of the *hlb*-converting prophage on membrane FAs likely reflects an interference between C16 and C18:2 incorporation. We further investigated the underlying mechanism.

### Incorporation of 1,3DG-derived C18:2 requires the kinase subunit FakB1 and is inhibited by C16

The substrate selectivity of the Fak kinase complex is determined by the FakB1 and FakB2 FA binding proteins, which are reported to bind saturated and unsaturated FAs, respectively (14, 15, 16). We confirmed these phenotypes using the USA300 *fakA*, *fakB1*, and *fakB2* mutants (Table 1) grown in media containing saturated and unsaturated free FAs (supplemental Fig. S5). When challenged with TG18:2, the *fakA* mutant did not incorporate C18:2, confirming that phosphorylation is required for the incorporation of C18:2 from TG18:2 into PLs (supplemental Fig. S6A, left). Interestingly, both *fakB1* and *fakB2* mutants failed to show selectivity; both mutants displayed similar capacities to incorporate C18:2 from TG18:2 (supplemental Fig. S6A, left). This indicates that while the incorporation of free C18:2 is FakB2-dependent, the incorporation from TG18:2 can be mediated by both FakB proteins.

**Fig. 4:**
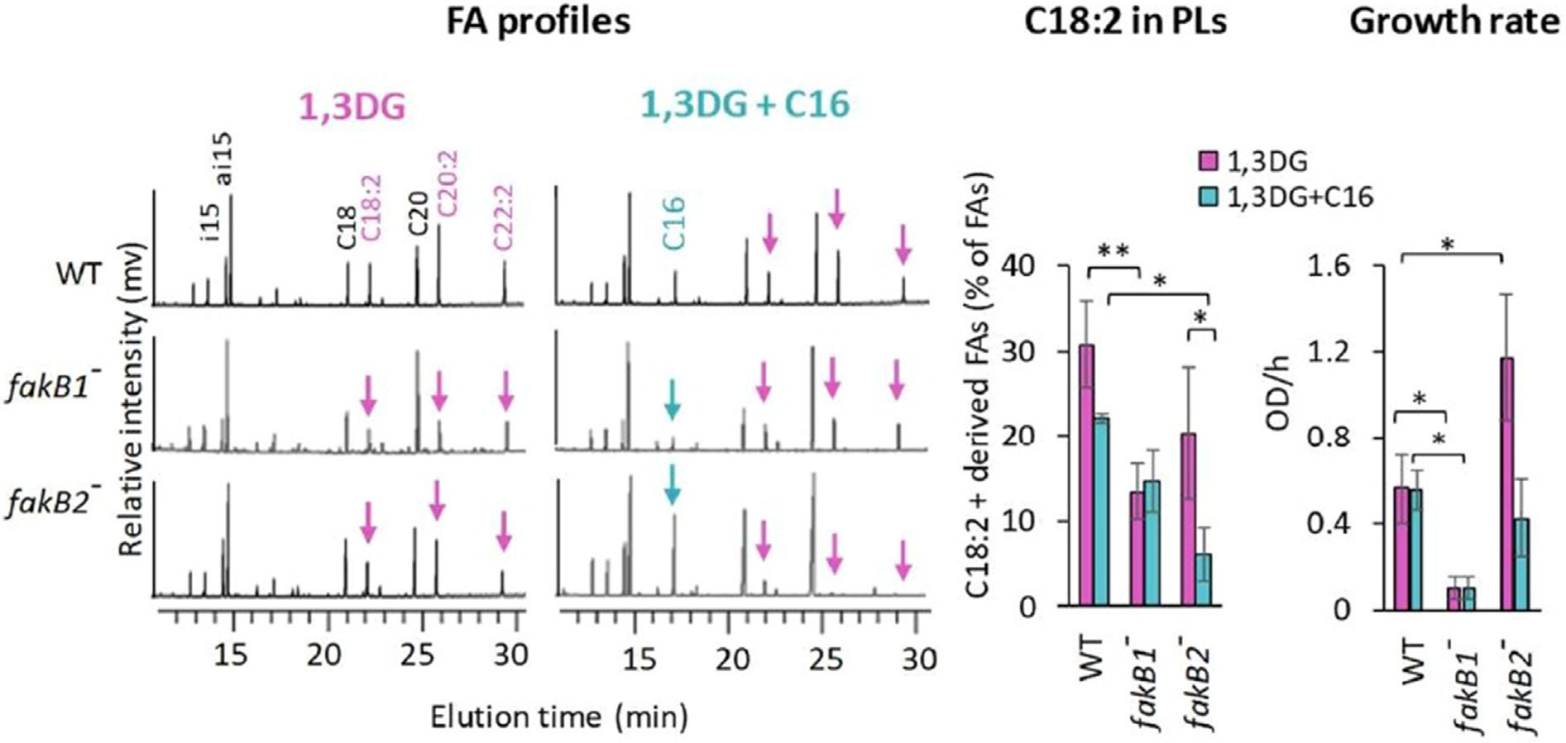
FakB1 mediates the incorporation of 1,3DG-derived C18:2, competing with C16 incorporation. USA300 (WT), *fakB1::tn*, and *fakB2::tn* mutant strains were cultured for 2 h in a BHI medium supplemented with 10 μM 1,3DG, with or without 20 μM C16. FA profiles were analyzed as described in Figure 1B. Bacterial growth and incorporated C18:2 were measured as in Figure 3A. In the FA profiles, purple arrows indicate C18:2 and its elongated forms (C20:2 and C22:2), blue arrows indicate C16. Growth is presented as OD_600_ per hour (OD/h). Data in histograms are means ± standard deviations from independent experiments (n = 5). Statistical significance was determined by the Mann-Whitney test for C18:2 incorporation and for OD_600_/h. ∗∗, *P* ≤ 0.01; ∗, *P* ≤ 0.05.

**Fig. 5:**
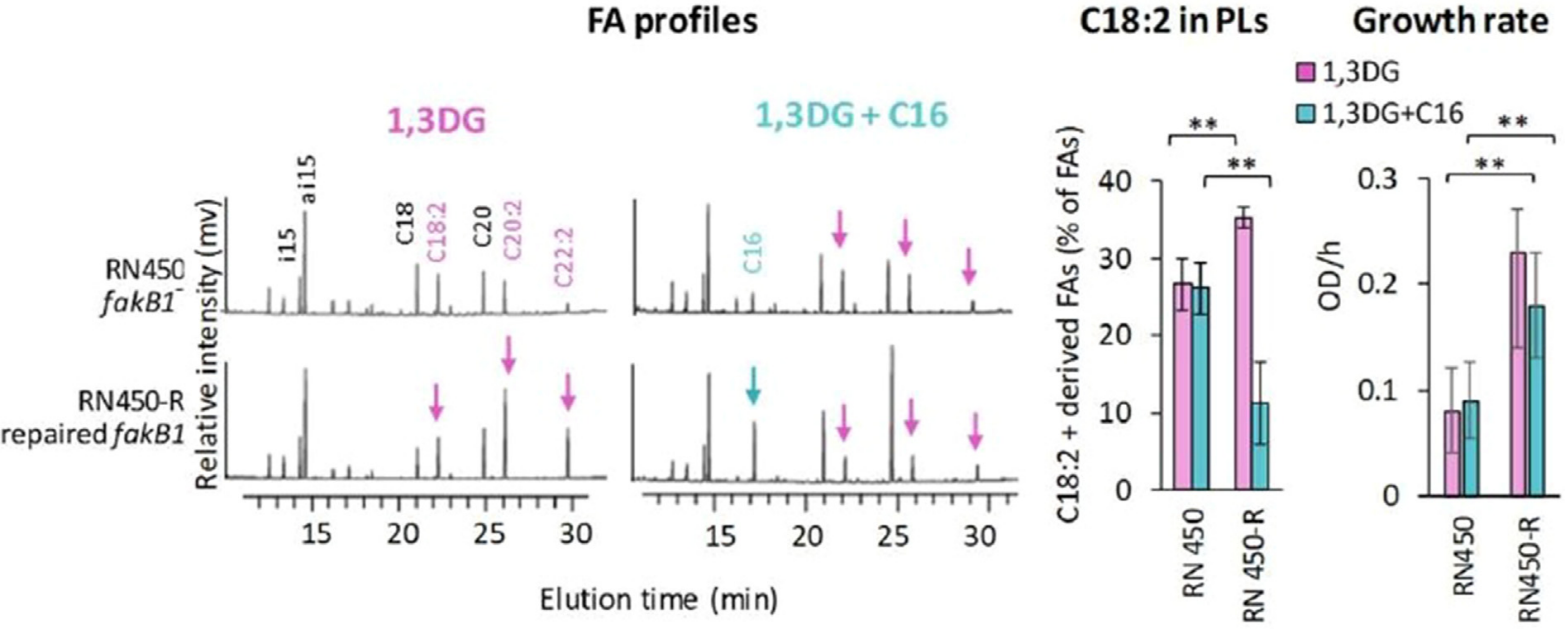
Restoration of *fakB1* enhances incorporation of 1,3DG-derived C18:2 and competition with C16. RN450 (*fakB1*^-^) and RN450-R (*fakB1*^+^) strains were cultured for 3 h in BHI medium supplemented with 10 μM 1,3DG, with or without 40 μM C16. FA profiles were determined as described in Figure 1B. Bacterial growth and incorporated C18:2 were measured as described in Figure 3A. In the FA profiles, purple arrows indicate C18:2 and elongated forms (C20:2 and C22:2). Blue arrows indicate C16. Growth is presented as OD_600_ per hour (OD_600_/h). Data in histograms are means ± standard deviations from independent experiments (n = 5). Statistical significance was determined using the Mann-Whitney test for C18:2 incorporation and OD_600_/h. ∗∗, *P* ≤ 0.01.

We further investigated whether the diacylglycerides released from TG18:2 by *S. aureus* lipases share this FakB binding flexibility. Compared to the TG18:2, incorporation of 1,2DG-derived C18:2 was significantly reduced in the *fakB2* mutant (supplemental Fig. S6A, right) aligning with the reported FakB2 binding preference for free unsaturated FAs (14, 15, 16). In contrast, incorporation of 1,3DG-derived C18:2 was higher in the *fakB2* mutant than in the *fakB1* mutant (Fig. 4). Moreover, the growth of the *fakB1* mutant was strongly impaired compared to the wild-type strain (Fig. 4). These results demonstrate that *fakB1* mediates the incorporation of 1,3DG-derived C18:2 and likely provides protection against the high toxicity of this diacylglycerol. This unexpected role of *fakB1* suggests competition with the sphingomyelin-derived C16, a known FakB1 ligand (14). To test this hypothesis, we added C16 in the presence of TG18:2 or 1,3DG. In both the USA300 WT and the *fakB1* mutant strains, the addition of free C16 resulted in a slight decrease in C18:2 incorporation from TG18:2 or 1,3DG (Fig. 4 and supplemental Fig. S6B). In stark contrast, the C16 addition to the *fakB2* mutant (which only has FakB1 available) almost completely halted the incorporation of C18:2 from both sources. Since 1,3DG is an intermediate product of TG18:2 hydrolysis, exogenous C16 may compete with 1,3DG-derived C18:2 for binding to FakB1. Interestingly, the protective effect of FakB1 against the incorporation of 1,3DG-derived C18:2 was not observed in the presence of C16 (Fig. 4, middle). The presence of C16 may lead to free C18:2, which is toxic to the bacteria and inhibits FASII activity (31), consistent with the observed effect of *hlb* on FA elongation (Fig. 3A, right). This may represent a form of toxicity not linked to the C18:2 incorporation into PLs. To confirm the direct role of *fakB1* in the incorporation of 1,3DG-derived C18:2, we used the RN450 strain, which carries an in-frame deletion in *fakB1*. This deletion affects two FA binding sites and the phosphotransfer reaction site (supplemental Fig. S1), resulting in a non-functional protein. This strain is cured of prophages and carries an intact *hlb* gene (Table 1). We constructed a derivative strain, designed RN450-R, in which *fakB1* is repaired (Table 1). As expected from the above results, 1,3DG-derived C18:2 was more efficiently incorporated by RN450-R than by the *fakB1*-defective RN450 strain (Fig. 5). In RN450, the presence of C16 had no effect on the incorporation of 1,3DG-derived C18:2, likely due to the absence of FakB1. In contrast, C16 strongly inhibited the incorporation of 1,3DG-derived C18:2 in the RN450-R strain (Fig. 5), confirming a direct role of *fakB1* in mediating the competition between 1,3DG-derived C18:2 and C16 for incorporation into PLs. C16 supplementation did not significantly affect the growth of RN450-R, suggesting strain-specific differences in the impact of competition on the toxicity of released C18:2. This strain is a derivative of NCTC8325, which was phage-cured using a two-step UV induction process, resulting in numerous mutations. However, these observations collectively identify *fakB1* as encoding the preferred binding protein for 1,3DG-derived C18:2 and as the key mediator of the competitive incorporation of C18:2 and C16.

### Inhibitors of FASII and *hlb* increase C18:2 incorporation and limit *S. aureus* adaptation

**Fig. 6:**
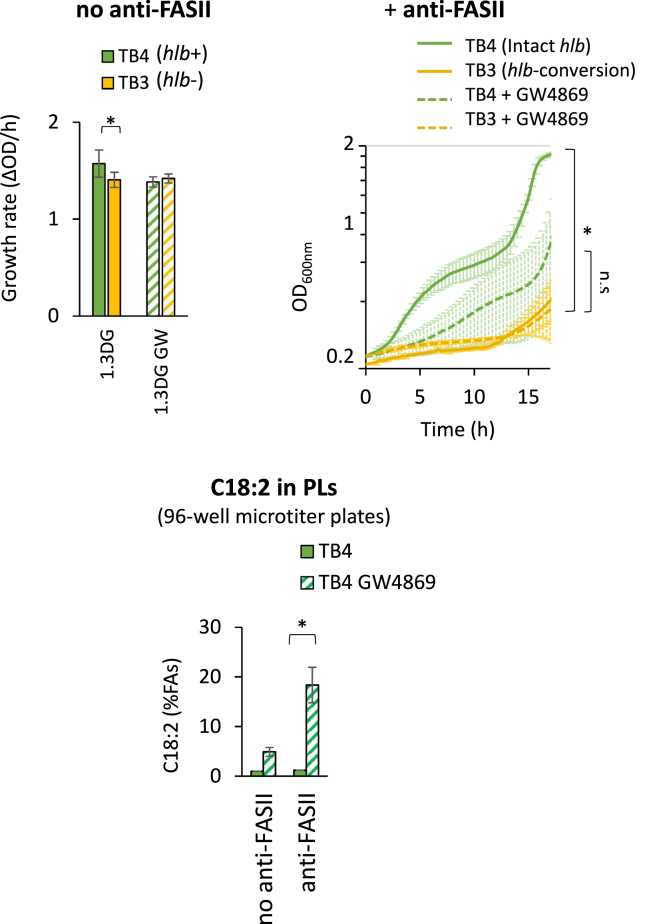
Hlb activity and anti-FASII treatments enhance C18:2 incorporation in *S. aureus* PLs. TB3 (фNM3 in *hlb*) and TB4 (no phage) strains were cultured in BHI medium supplemented with 10% mouse serum, 30 μM 1,3DG, with or without 0.5 μg/ml AFN-1252 (anti-FASII), and with or without 50 μM GW4869 (inhibitor of Hlb). Top left: bacterial growth rates in the absence of anti-FASII treatment, performed in glass tubes. Top right: growth kinetics in the presence of AFN-1252, measured in 96-well microtiter plates with OD600 reading every 10 min. Bottom: C18:2 incorporation with or without AFN-1252. C18:2 incorporation was determined in the TB4 and TB3 strains as described in Figure 2B. Data are means ± standard deviations from independent experiments (n = 4). Statistical significance was determined by the Mann-Whitney test for on OD_600_/h and by the Wilcoxon Signed Rank test for incorporated C18:2; ∗, *P* ≤ 0.05. n.s: not significant.

In the presence of an antibiotic targeting FASII (anti-FASII), *S. aureus* adapts by incorporating exogenous FAs, including unsaturated FAs (8). Consequently, bacterial growth becomes exclusively reliant on exogenous FAs under these conditions, likely increasing levels and toxicity of PUFAs in the membrane. We therefore analyzed the potential synergistic effects of inhibiting both FASII and Hlb activities on *S. aureus* growth. AFN-1252 was used as the FASII inhibitor; this drug targets FASII enoyl reductase enzyme FabI and is effective and safe against skin infections (50, 51). GW4869 was used as the sphingomyelinase inhibitor, as it inhibits *S. aureus* sphingomyelinase activation (39, 40). Growth of TB3 and TB4 isogenic strains (Table 1) was compared in BHI-mouse serum medium supplemented with 1,3DG, with or without GW4869. When FASII was active, TB3 (*hlb*-converted) exhibited slightly decreased growth compared to TB4 (intact *hlb*), and GW4869 reversed the growth advantage of TB4 (Fig. 6, top left). The addition of the FASII inhibitor AFN-1252 significantly slowed growth. As previously reported, *S. aureus* adapted to AFN-1252 in the presence of serum (8) but the TB4 strain displayed a markedly shortened latency period prior to adaptation compared to TB3 (outgrowth <2 h instead of 12 h) (Fig. 6, top right). Furthermore, the addition of GW4869 did not significantly affect TB3 strain growth but delayed that of TB4. These results demonstrate that Hlb activity limits the inhibitory effects of AFN1252. We determined FA profiles from these cultures and analyzed the percentage of C18:2 in PLs. AFN-1252 increased the proportion of C18:2 in the PLs of the TB4 strain, especially in the presence of GW4869. We also noticed that AFN-1252 treatment increased the C18:2 percentage fourfold (Fig. 6, bottom), suggesting a synergistic effect on C18:2 toxicity in the *S. aureus* membrane. These results suggest that Hlb activity accelerates adaptation to anti-FASII by limiting C18:2 incorporation and toxicity in the membrane. Safety in humans has been previously demonstrated for both the anti-FASII antibiotic AFN1252 and the Hlb inhibitor GW4869 (50, 52), suggesting that they may be good candidates for combinatory strategies against *S. aureus*.

## Discussion

**Fig. 7:**
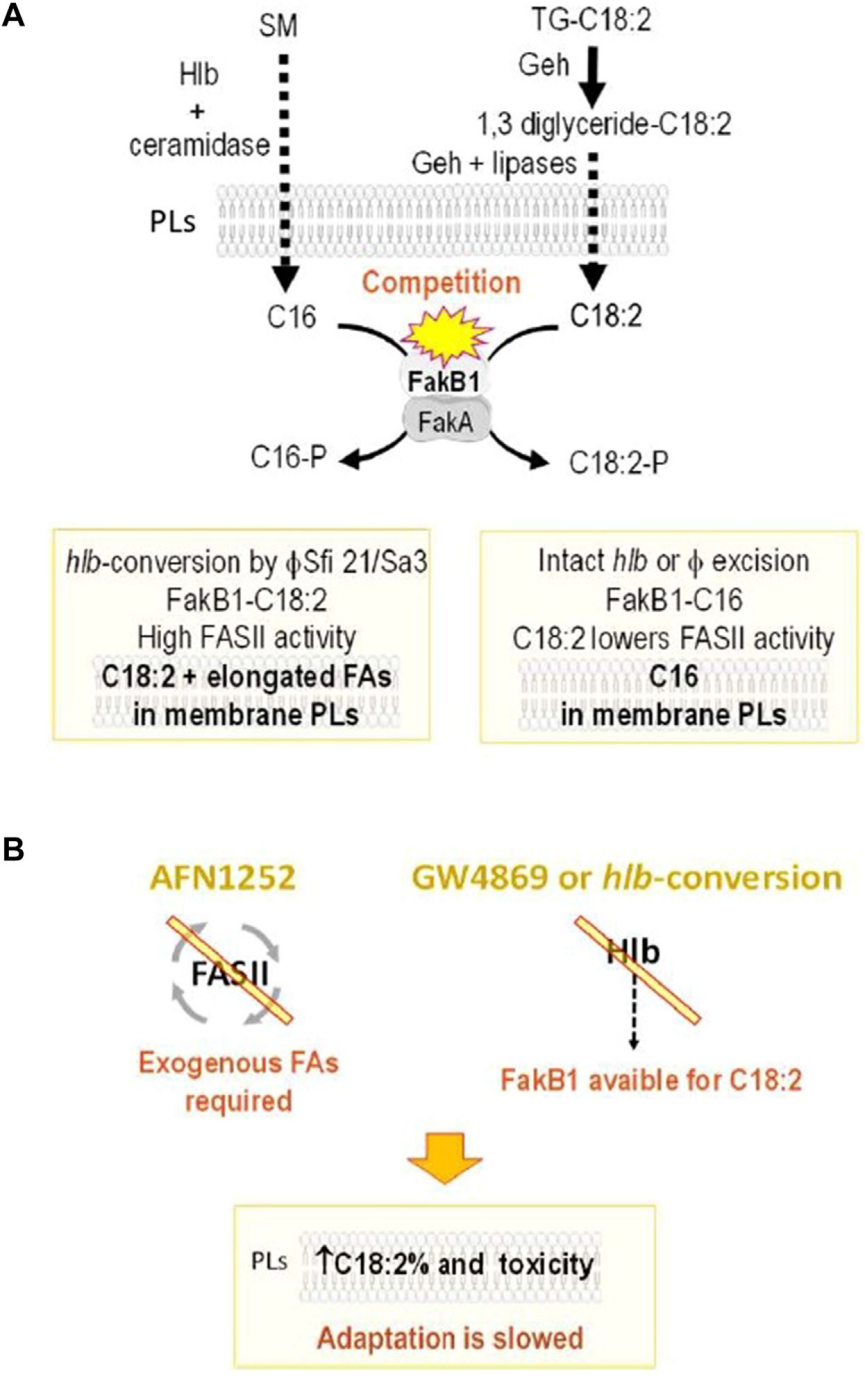
Mechanistic model of C18:2 toxicity in *S. aureus* and its enhancement by inhibition of FASII or sphingomyelinase activity. A: Modulation of C18:2 incorporation by the *hlb*-converting prophage is driven by FA competition for FakB1. The model highlights three key features: 1) The 1,3-diacylglycerol intermediate, released by the *S. aureus* Geh lipase, increases the incorporation and toxicity of C18:2 in the bacterial membrane. 2) C18:2 derived from 1,3-diacylglycerol binds to FakB1 for incorporation into PLs, in contrast to free C18:2 or that derived from 1,2-diacylglycerol. 3) Hlb activity promotes the release of sphingomyelin-derived C16, which competes with C18:2 for FakB1 binding, thereby preventing C18:2 incorporation into PLs. Unbound C18:2 inhibits FASII (31), impairing FA elongation and bacterial growth. As an active lysogen, the prophage rapidly regulates Hlb activity (23, 26). Depending on the availability of Hlb substrates in the environment, it fine-tunes bacterial adaptation to the highly toxic C18:2 derived from 1,3-diacylglycerol. B: Blocking FASII or sphingomyelinase activity exacerbates C18:2 incorporation, further impairing bacterial adaptation. Under anti-FASII conditions, S *aureus* relies on exogenous FAs for growth (8), such as C18:2, which is incorporated into membrane PLs and is highly toxic. Moreover, inhibiting Hlb activity reduces competition for FakB1 binding, enabling increased C18:2 incorporation into PLs (see Fig. 7A). Thus, inhibitors targeting FASII or Hlb are potential combinatory drugs that could potentiate the natural defense mechanism mediated by C18:2. SM: sphingomyelin, TG-C18:2: triglyceride containing C18:2, 1,3 diglyceride-C18:2: 1,3-diacylglycerol containing C18:2.

This study highlights the crucial role of *hlb*-converting prophages and Hlb sphingomyelinase activity in the lipid metabolism of *S. aureus* and its adaptation to the presence of an anti-FASII agent (Fig. 7). We first demonstrated that Hlb activity unexpectedly inhibits C18:2 incorporation into the bacterial membrane. This effect was linked to the FakB1 kinase subunit, as FAs released from TGs and sphingomyelins compete for this function (Fig. 7A). This mechanism involves a TG-derived 1,3-diacylglycerol, which promotes significant C18:2 incorporation via FakB1 binding, competing with sphingomyelin-derived C16. FakB1’s affinity for C18:2, specifically derived from 1,3DG (not free C18:2 or that derived from 1,2DG) remains to be fully elucidated. The Hlb/C18:2/FakB1 connection may represent a natural Achilles' heel exploited by Sfi 21/Sa3 prophages to modulate bacterial fitness in response to the toxicity of this PUFA.

TGs are the main constituents of human fat and blood, serving as key circulating reservoirs of C18:2 (53). During infection, both Geh and Hlb lipases are active (27, 54). We speculate that C18:2/FakB1 binding and its competition with sphingomyelin-derived C16 may occur in vivo. Sfi21/Sa3 are *hlb-*converting prophages that act as regulatory switches for Hlb in most clinical isolates (21, 22, 23, 24, 27, 55). Various stressors, as H_2_O_2_ or biocides, induce prophage excision into extrachromosomal episomes, allowing rapid phage excision/reinsertion, and control of Hlb activity (23, 26). As active lysogens, these prophages may reversibly control the Hlb/C18:2/Fakb1 interaction. This mechanism offers novel insights into the prevalence of *hlb*-converting prophages in clinical isolates and the role of the lipid environment in *S. aureus* adaptation during infection. The incorporation of PUFAs such as C18:2 into PLs alters essential host-interaction functions, including biofilm formation, virulence factor secretion, and interference with immune defense (19, 20, 56, 57, 58). Additionally, C18:2 and sphingolipid metabolisms are critical pathways that *S. aureus* manipulates to impair macrophage efficacy (59). Thus, the control of the Hlb/C18:2/FakB1 interaction by *hlb*-converting prophages may play a significant role in *S. aureus* evasion of innate immunity.

The results demonstrate that the levels and balance of TGs and sphingomyelins, along with FA composition and their position in TGs, are crucial factors for *S. aureus* fitness. TGs and sphingomyelins vary based on the state of infection, the infected site, and individual factors such as diet and genetic makeup (53, 59, 60, 61, 62). Interestingly, several diseases associated with a high risk of infection or severe evolution, such as in Crohn’s disease, type 2 diabetes, obesity, and cystic fibrosis, exhibit dysbiosis in TGs and/or sphingomyelins (44, 53, 59, 63, 64, 65, 66, 67, 68, 69). Furthermore, novel strategies are particularly needed to combat sepsis (70, 71). Lipid emulsions are approved or under investigation for intravenous nutrition in sepsis patients ((https://clinicaltrials.gov/study/NCT03405870) (72)). The mechanism connecting TGs and SMs opens new hypotheses and potential future lipid formulations for prevention or to counter fatal outcomes.

This study also demonstrates that *hlb*-conversion limits adaptation to anti-FASII treatment (mechanistic model in Fig. 7B). In line with the Hlb/C18:2/FakB1 connection, the absence of Hlb activity increases the incorporation of toxic C18:2, thereby delaying adaptation to anti-FASII treatment. What insights can be drown from previous in vivo studies of anti-FASII adaptation? *S. aureus* was shown to adapt efficiently to anti-FASII treatment in a mouse model (8). Given that Hlb activity is known to occur during infection (for review (25)), we propose that prophage excision facilitates anti-FASII adaptation in vivo. Moreover, the composition of membrane fatty acids has been shown to influence the efficacy of other antibiotics, such as daptomycin and vancomycin (73, 74). This highlights the potential relevance of the Hlb/C18:2/FakB1 connection for future drug screening and development. Furthermore, both FASII and Hlb inhibitors increase the proportion of C18:2 in membrane PLs. Consequently, combining an Hlb inhibitor or anti-FASII treatment with TG18:2 enrichment could have a synergistic effect against *S. aureus*. A strategy incorporating this natural lipid-based mechanism may help reduce drug dosages and lower the risk of resistant variant emergence. In conclusion, the mechanism by which these prophages alter *S. aureus* lipid metabolism is pivotal for addressing challenges in infection prevention and healthcare. These findings highlight the importance of targeting the Hlb pathway and lipid balance, particularly the diacylglycerols/sphingomyelins ratio, as a pomising avenue for the development of novel therapeutic strategies.

## Data availability

All data supporting the findings of this study are available within this article and its supplemental data.

## Supplemental data

This article contains supplemental data.

## Conflict of interest

The authors declare that they have no conflicts of interest with the contents of this article.

## Figures and Tables

**Supplementary Figure S2:**