# Supplementary material for: Prophages divert Staphylococcus aureus defenses against host lipids
Source: J Lipid Res. 2024 Nov 5;65(12):100693. doi: 10.1016/j.jlr.2024.100693 (PMC11721228; doi:10.1016/j.jlr.2024.100693)
Supplement: Supplementary Figure S3 [file mmc1.pdf]

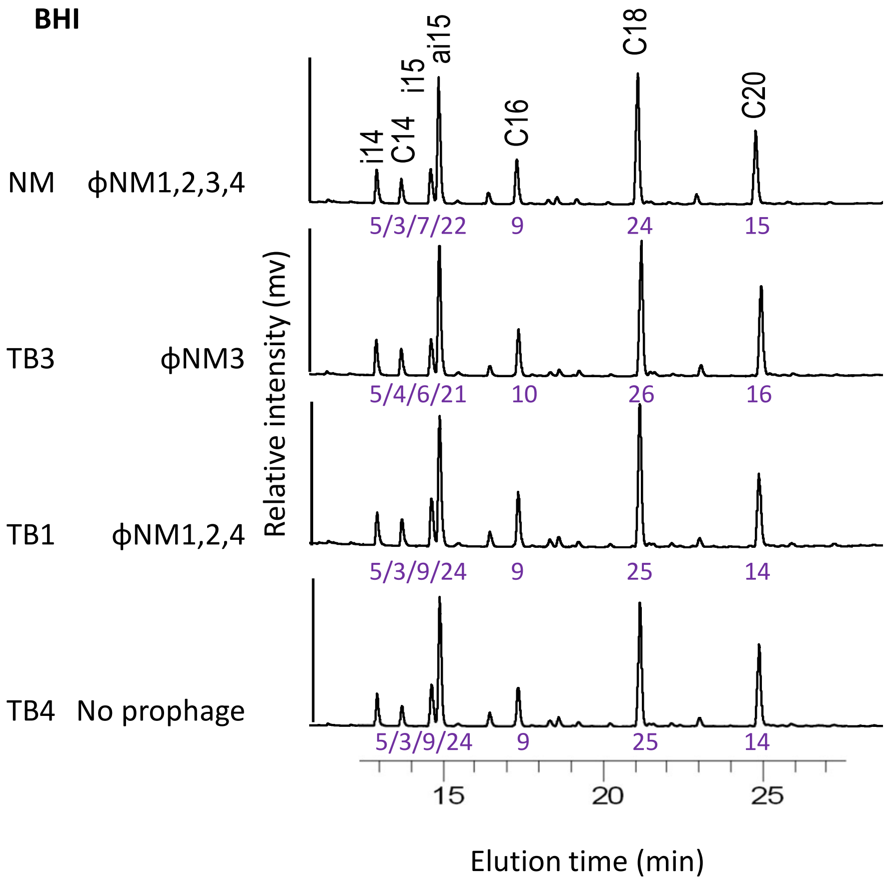

Supplementary Figure S3. FA profiles of *S. aureus* cultured in BHI medium are not affected by the prophage status. Four isogenic Newman strains, either lysogenized or not by prophages (ϕNM1, ϕNM2, ϕNM3, ϕNM4) as shown, were cultured in BHI. FAs were extracted and analyzed as described in Fig. 1B. Representative FA profiles and means of FA relative amounts (in purple) from three independent experiments are shown. Statistical significance was determined by the Kruskal-Wallis test to compare the FA compositions between the four strains. Profiles were not significantly different, p value> 0.05.
