# Supplementary material for: Prophages divert Staphylococcus aureus defenses against host lipids
Source: J Lipid Res. 2024 Nov 5;65(12):100693. doi: 10.1016/j.jlr.2024.100693 (PMC11721228; doi:10.1016/j.jlr.2024.100693)
Supplement: Supplementary Figure S4 [file mmc2.pdf]

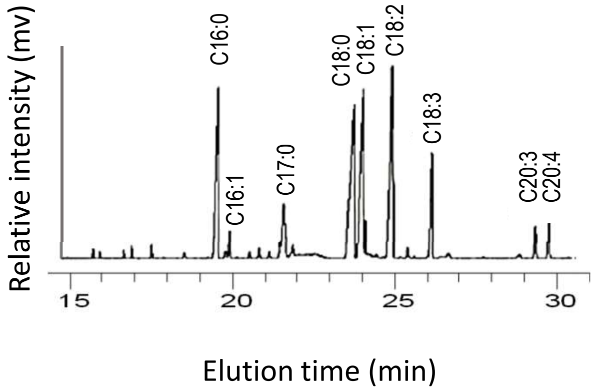

Supplementary Figure S4. Fatty acid profile from adult bovine serum. A representative profile, determined as in Figure 1B is shown. This serum contains different PUFAs: linoleic acid (C18:2), α-linolenic acid (C18:3 ω-3), dihomo-γ-linolenic acid (20:3 ω−6), and arachidonic acid (C20:4 ω-6).
