# Supplementary material for: Prophages divert Staphylococcus aureus defenses against host lipids
Source: J Lipid Res. 2024 Nov 5;65(12):100693. doi: 10.1016/j.jlr.2024.100693 (PMC11721228; doi:10.1016/j.jlr.2024.100693)
Supplement: Supplementary Figure S5 [file mmc3.pdf]

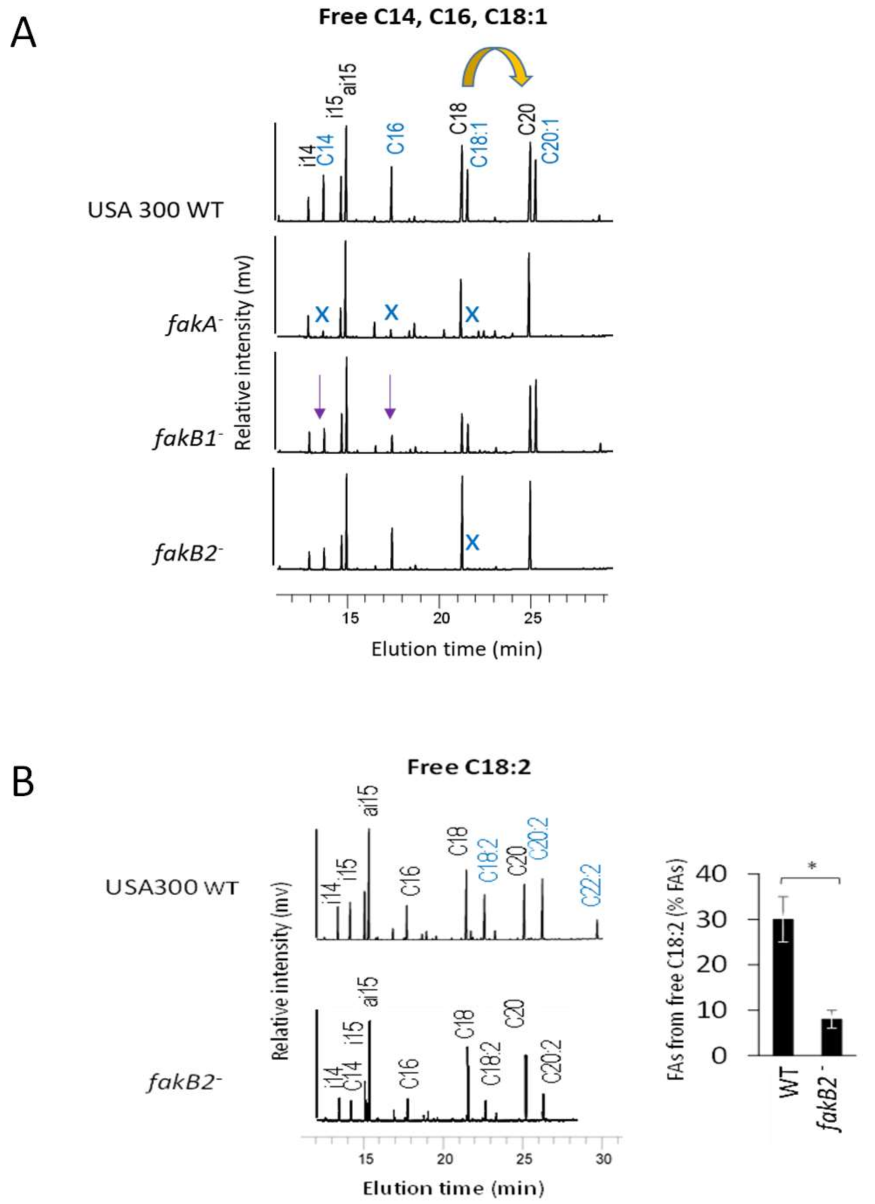

Supplementary Figure S5. Phenotype validation of *fakA, fakB1* and *fakB2* mutants. The USA300 wild type (WT) and mutant strains were cultured in BHI medium in the presence of exogenous FAs. Total membrane FAs were extracted, analysed, and presented as in Fig. 1B. (A) FAs profiles from the WT strain and the *fak* mutants cultured in the presence of an FA cocktail (free C14:0, C16:0, and C18:1, 0.17 mM each). The three exogenous FAs and their elongated forms are indicated in blue. The orange arrow indicates C18:1 elongation into C20:1. Blue crosses indicate that *fakA* is essential for FA incorporation and *fakB2* is essential for C18:1 incorporation. Purple arrows indicate that *fakB1* is involved in the incorporation of C14 and C16. (B) *fakB2* is required for the incorporation of free C18:2. The WT strain and the *fakB2*::tn mutant were cultured in the presence 10µM free C18:2. Left: FA profiles, C18:2 and its elongated forms are represented in blue. Right: C18:2 incorporation into PLs. Note that free C18:2 is poorly incorporated in the *fakB2* mutant compared to the WT. Data presented are means +/- standard deviations from independent experiments (n=3). Statistical significance was determined by the Mann-Whitney test. *, p≤0.05.
