# Supplementary material for: Prophages divert Staphylococcus aureus defenses against host lipids
Source: J Lipid Res. 2024 Nov 5;65(12):100693. doi: 10.1016/j.jlr.2024.100693 (PMC11721228; doi:10.1016/j.jlr.2024.100693)
Supplement: Supplementary Figure S6 [file mmc4.pdf]

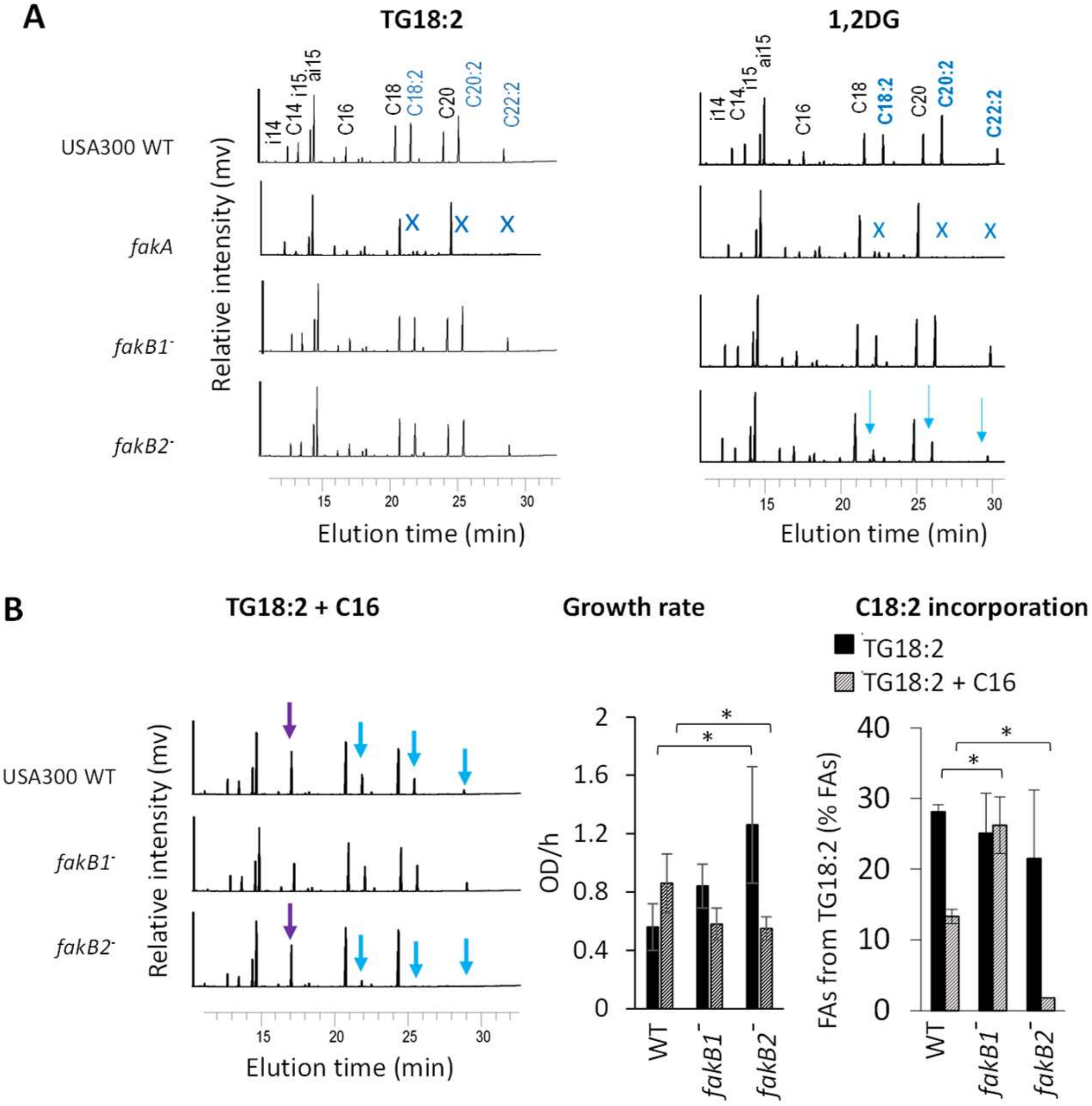

- **Supplementary Figure S6.** Roles of *fak* genes in the incorporation of C18:2 from TG18:2 or 1,2DG. Experiments and FAs profiles were performed as described in Figure 4, except that the USA300 WT and *fak* mutant strains (Table 1) were cultured in the presence of 30µM TG18:2 or 1,2DG, as sources of C18:2. (A) Representative FA profiles from *fakA, fakB1* and *fakB2* mutants cultured in the presence of TG18:2 or 1,2DG. Left: The *fakA* defective strain fails to incorporate C18:2 from TG18:2, while both *fakB1* and *fakB2* mediate incorporation of TG18:2-derived C18:2. Right: The *fakB2* gene, and not *fakB1*, affects C18:2 incorporation from 1,2 dilinolein (1,2DG). (B) C16 inhibits the incorporation of C18:2 from TG18:2 through competition for FakB1. The *fakB2* mutant (encoding only *fakB1*) fails to incorporate TG18:2-derived C18:2 in the presence of C16. Growth rates presented as OD_{600} per hour suggest that FakB1 protects against C18:2 toxicity and that C16 inhibits growth when only FakB1 is active by releasing free C18:2 in *S. aureus*. TG18:2-derived C18:2 and its elongated forms are indicated in blue, and blue crosses indicating their absence. Purple arrows indicate a C16 increase due to exogenous supply. Blue arrows indicate the inhibition of TG18:2-derived C18:2 and its elongated forms. Profiles are representative of independent experiments (n=3). Histograms are means +/- standard deviations from these experiments. Statistical significance was determined by the Mann-Whitney test.; *, $p \leq 0.05$.
